# Mechanisms of the IAA and ACC-deaminase producing strain of *Trichoderma longibrachiatum* T6 in enhancing wheat seedling tolerance to NaCl stress

**DOI:** 10.1186/s12870-018-1618-5

**Published:** 2019-01-11

**Authors:** Shuwu Zhang, Yantai Gan, Bingliang Xu

**Affiliations:** 1 Gansu Provincial Key Laboratory of Arid Land Crop Science, Gansu Agricultural University/College of Plant protection, Gansu Agricultural University/ Biocontrol Engineering Laboratory of Crop Diseases and Pests of Gansu Province, Lanzhou, 730070 China; 2 Agriculture and Agri-Food Canada/Government of Canada Swift Current Research & Development Centre, Swift Current, Saskatchewan SK S9H 3X2 Canada

**Keywords:** *Trichoderma* species, Salt stress, Wheat seedling, Plant growth promotion, 1-aminocyclopropane-1-carboxylate-deaminase, Indole acetic acid, Ionic toxicity, Gene expression

## Abstract

**Background:**

*Trichoderma* species, a class of plant beneficial fungi, may provide opportunistic symbionts to induce plant tolerance to abiotic stresses. Here, we determined the possible mechanisms responsible for the indole acetic acid (IAA) and 1-aminocyclopropane-1-carboxylate-deaminase (ACC-deaminase) producing strain of *Trichoderma longibrachiatum* T6 (TL-6) in promoting wheat (*Triticum aestivum* L.) growth and enhancing plant tolerance to NaCl stress.

**Results:**

Wheat treated with or without TL-6 was grown under different levels of salt stress in controlled environmental conditions. TL-6 showed a high level of tolerance to 10 mg ml^− 1^ of NaCl stress and the inhibitory effect was more pronounced at higher NaCl concentrations. Under NaCl stress, the activity of ACC-deaminase and IAA concentration in TL-6 were promoted, with the activity of ACC-deaminase increased by 26% at the salt concentration of 10 mg ml^− 1^ and 31% at 20 mg ml^− 1^, compared with non-saline stress; and the concentration of IAA was increased by 10 and 7%, respectively (*P* < 0.05). The increased ACC-deaminase and IAA concentration in the TL-6 strain may serve as an important signal to alleviate the negative effect of NaCl stress on wheat growth. As such, wheat seedlings with the ACC-deaminase and IAA producing strain of TL-6 treatment under NaCl stress increased the IAA concentration by an average of 11%, decreased the activity of ACC oxidase (ACO) by an average of 12% and ACC synthase (ACS) 13%, and decreased the level of ethylene synthesis and the content of ACC by 12 and 22%, respectively (*P* < 0.05). The TL-6 treatment decreased the transcriptional level of ethylene synthesis genes expression, and increased the IAA production genes expression significantly in wheat seedlings roots; down-regulated the expression of ACO genes by an average of 9% and ACS genes 12%, whereas up-regulated the expression of IAA genes by 10% (*P* < 0.05). TL-6 treatments under NaCl stress decreased the level of Na^+^ accumulation; and increased the uptake of K^+^ and the ratio of K^+^/Na^+^, and the transcriptional level of Na^+^/H^+^ antiporter gene expression in both shoots and roots.

**Conclusions:**

Our results indicate that the strain of TL-6 effectively promoted wheat growth and enhanced plant tolerance to NaCl stress through the increased ACC-deaminase activity and IAA production in TL-6 stain that modulate the IAA and ethylene synthesis, and regulate the transcriptional levels of IAA and ethylene synthesis genes expression in wheat seedling roots under salt stress, and minimize ionic toxicity by disturbing the intracellular ionic homeostasis in the plant cells. These biochemical, physiological and molecular responses helped promote the wheat seedling growth and enhanced plant tolerance to salt stress.

## Background

*Trichoderma* spp., a class of soil-borne fungi, is considered a potential bio-control agent effective against plant pathogens and plant parasitic nematodes [1, 2]. The microorganism often found in rhizsphere can provide beneficial effects on plant growth and yields [3]. The mechanism of *Trichoderma* spp. promoting plant growth is not clear, but a number of studies with different microorganisms show that some metabolic processes and pathways may be involved. For example, auxin plays an important role in root architecture configuration in association with *Trichoderma* spp. [4]; the strain *T. asperellum* T203 produces ACC-deaminase that regulates the endogenous ACC level and stimulates root elongation [5] and enhances plant tolerance to abiotic stress [6]; the strain *T. virens* Gv. 29–8 promotes *Arabidopsis* growth through auxin response pathway to modulate root development and activate auxin regulated gene expression [4]. Furthermore, plants roots colonized by *T. harzianum* increased the level of antioxidant enzymes that helped enhance plant resistance to abiotic stresses [7–9]. However, little is known about the synthesis of IAA and ACC-deaminase in *Trichoderma longibrachiatum* T6 (TL-6) that promotes plant growth and enhances plant tolerance to salt stress. It is unknown whether the function of TL-6 in promoting plant growth and enhancing plant tolerance can be retained under salt stress.

Salinity is one of the important abiotic stresses that limit plant growth and crop yield [10–12]. Globally, saline soil accounts for more than 7% of the total arable land and the trend of soil salinization has been increasing in recent years [13]. In China, the area of saline soil is greater than 100 million hectare, accounting for about 37% of the total arable land in the country [14]. In saline soil, plants experience dehydration, nutrient deficiency, membrane dysfunction, and metabolic and photosynthetic activity reduction [13, 15, 16]. To decrease the negative effects of salt stress on plant growth and development, large efforts have been taken in developing salt tolerant plant genotypes through conventional breeding or genetic engineering. However, those efforts have shown limited success as the functional genes responsible for salt tolerance can be lost easily in transgenic plants [17].

An alternative strategy to improve plant tolerance to salt stress is the use of plant growth promoting microbes. Arbuscular mycorrhizal fungi have been reported to enhance the ability of plants to cope with salinity [18–20]. The colonization of arbuscular mycorrhizal fungi helps modulate the ROS-scavenging system in salt-stressed wheat [21]. Also, some *Trichoderma* species can directly colonize plant roots and stimulate roots growth, and thus, enhance plant tolerance to abiotic stresses [22]. The isolate of *T. harzianum* was found to help mitigate NaCl stress in mustard (*Brassica juncea* L.) through antioxidative defense system [23]. Seed biopriming with the isolate of *T. harzianum* alleviated the negative effects of salinity stress in wheat [11]. In a previous study, we found that application of TL-6 improved wheat tolerance to salt stress [24]. However, our previous study was unable to determine the possible mechanisms responsible for TL-6 promoting wheat seedling growth and enhancing plant tolerance to salt stress, and little is known about whether such function of the TL-6 strain can be retained under different levels of salt stress.

The present study was to test the hypothesis that the TL-6 strain enhancing wheat seedling tolerance to salt stress is through (i) the synthesis of IAA and ACC-deaminase in TL-6 that regulate wheat tolerance to NaCl stress, (ii) the increased IAA concentration and the enhanced gene expression of transcriptional levels of IAA synthesis, and the decreased ethylene synthesis and the down-regulated gene expression of transcriptional levels of ethylene synthesis in wheat with the TL-6 treatment under NaCl stress, and (iii) the increased Na^+^ extrusion and the enhanced gene expression of transcriptional level of Na^+^/H^+^ antiporter in wheat by maintaining lower Na^+^/K^+^ ratio in wheat that stimulates seedling growth with the application of TL-6 under NaCl stress. These determinations will allow an assessment of the mechanisms responsible for TL-6 promoting wheat growth and improving the tolerance to NaCl stress.

## Results

### Effect of NaCl stress on the colony diameter, spores production and mycelia weight of TL-6 strain

**Fig. 1:**
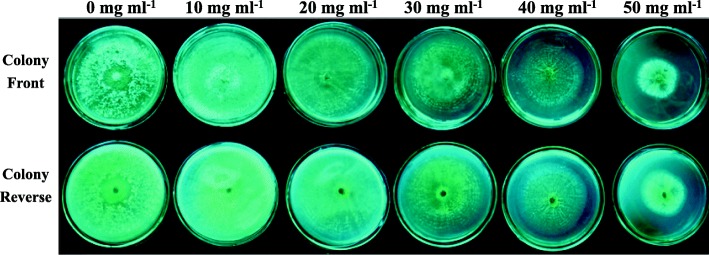
Colony growth of *Trichoderma longibrachiatum* T6 under the different (0, 10, 20, 30, 40, and 50 mg ml^− 1^) concentrations of NaCl solutions 7 days after treatment at 25 °C

Measurement of the effect of NaCl stress on the growth of TL-6 strain, our results showed that the NaCl stress treatment had a significant impact on the colony diameter, spores production and mycelia weight of TL-6 strain (Table 1 and Fig. 1). At Days 6 and 7 of salt treatment, the TL-6 strain tolerated the 0, 10 and 20 mg ml^− 1^ of NaCl stress treatments, but the differential inhibitory effects were observed with the salt concentrations increased to 30, 40 and 50 mg ml^− 1^ where the NaCl treatments significantly decreased the TL-6 growth (*P* < 0.05). At Day 7, the number of spores produced by TL*-*6 was significantly higher when treated with the 10 mg ml^− 1^ of NaCl solution compared with 0 mg ml^− 1^ of NaCl concentration, but was significantly lower at the concentrations of NaCl greater than 20 mg ml^− 1^ (Table 1). Also, increased concentrations of NaCl decreased the dry weight of mycelia significantly at salt concentrations greater than 20 mg ml^− 1^ (Table 1).

**Table 1:** Effect of different concentrations of NaCl solutions on the growth of *Trichoderma longibrachiatum* T6

Salt concentrations (mg ml^− 1^)	Days in incubation (d)					Number of spores produced (10^6^ spores ml^− 1^)	Mycelia dry weight (g)
	3	4	5	6	7		
	Colony diameter (cm)						
0	6.8 ± 0.2^a^	9.0 ± 0.0^a^	9.0 ± 0.0^a^	9.0 ± 0.0^a^	9.0 ± 0.0^a^	27.3 ± 1.2^b^	0.193 ± 0.010^a^
10	5.0 ± 0.3^ab^	7.3 ± 0.2^ab^	9.0 ± 0.0^a^	9.0 ± 0.0^a^	9.0 ± 0.0^a^	29.3 ± 1.1^a^	0.192 ± 0.007^a^
20	3.8 ± 0.3^b^	5.5 ± 0.3^b^	8.4 ± 0.2^a^	9.0 ± 0.0^a^	9.0 ± 0.0^a^	15.0 ± 0.9^c^	0.175 ± 0.008^b^
30	2.4 ± 0.2^c^	3.6 ± 0.3^c^	5.3 ± 0.3^b^	6.5 ± 0.3^b^	7.1 ± 0.3^b^	4.5 ± 0.2^d^	0.123 ± 0.003^c^
40	1.4 ± 0.1^cd^	1.9 ± 0.2^d^	2.5 ± 0.2^c^	3.0 ± 0.1^c^	3.5 ± 0.3^c^	0.1 ± 0.02^e^	0.086 ± 0.005^d^
50	0.9 ± 0.04^d^	1.4 ± 0.2^d^	1.8 ± 0.3^c^	2.1 ± 0.2^c^	2.7 ± 0.2^d^	0.07 ± 0.01^e^	0.076 ± 0.003^d^

Data are mean ± standard error of replicates, and the number of spore production was determined 7 days after treatment, the mycelia dry weight was determined 5 days after inoculation. Different letters in the same column mean significant difference at the *P* < 0.05 level by Duncan’s new multiple range test (*n* = 12)

### Determination of IAA production and ACC-deaminase activity in TL-6

**Fig. 2:**
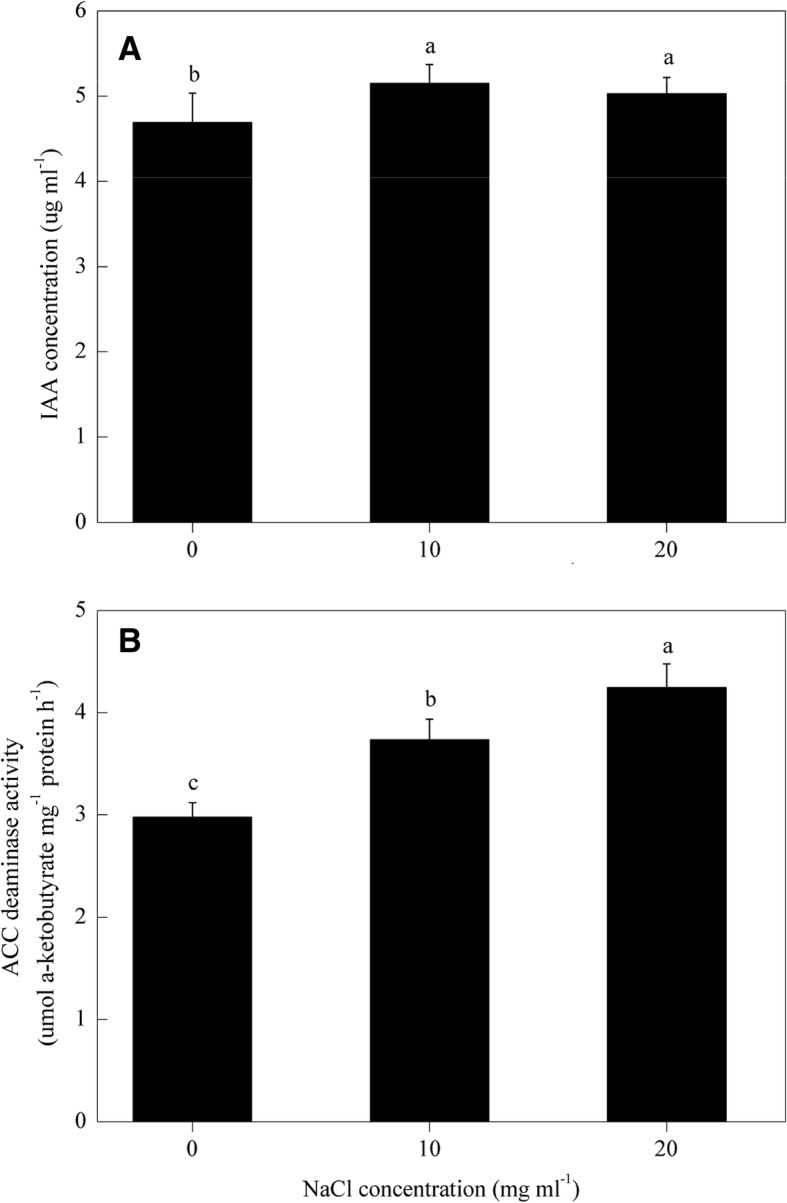
Effect of different concentrations of NaCl solutions on (**a**) IAA concentration and (**b**) the activity of ACC-deaminase in *Trichoderma longibrachiatum* T6. The line bars represent the standard errors of the means. Different letters denote significant difference at the *P* < 0.05 level by Duncan’s new multiple range test (*n* = 12)

The concentration of IAA and the activity of ACC-deaminase in TL-6 were determined under different levels of NaCl concentrations. The strain of TL-6 produced both IAA (Fig. 2a) and ACC-deaminase (Fig. 2b) regardless of NaCl concentration. However, the amounts of IAA produced by TL-6 at the NaCl concentrations of 10 and 20 mg ml^− 1^ were significantly higher (by 10 and 7%) compared with 0 mg ml^− 1^ of NaCl concentration (Fig. 2a) (*P* < 0.05). In contrast to IAA, the activity of ACC-deaminase differed significantly with the NaCl concentration (Fig. 2b) (*P* < 0.05). Compared with 0 mg ml^− 1^ of NaCl concentration, the NaCl treatment at 10 mg ml^− 1^ increased the activity of ACC-deaminase by 26%, and the doubling NaCl concentration to 20 mg ml^− 1^ increased the activity of ACC-deaminase by 31% (*P* < 0.05).

### IAA production in wheat seedling

**Fig. 3:**
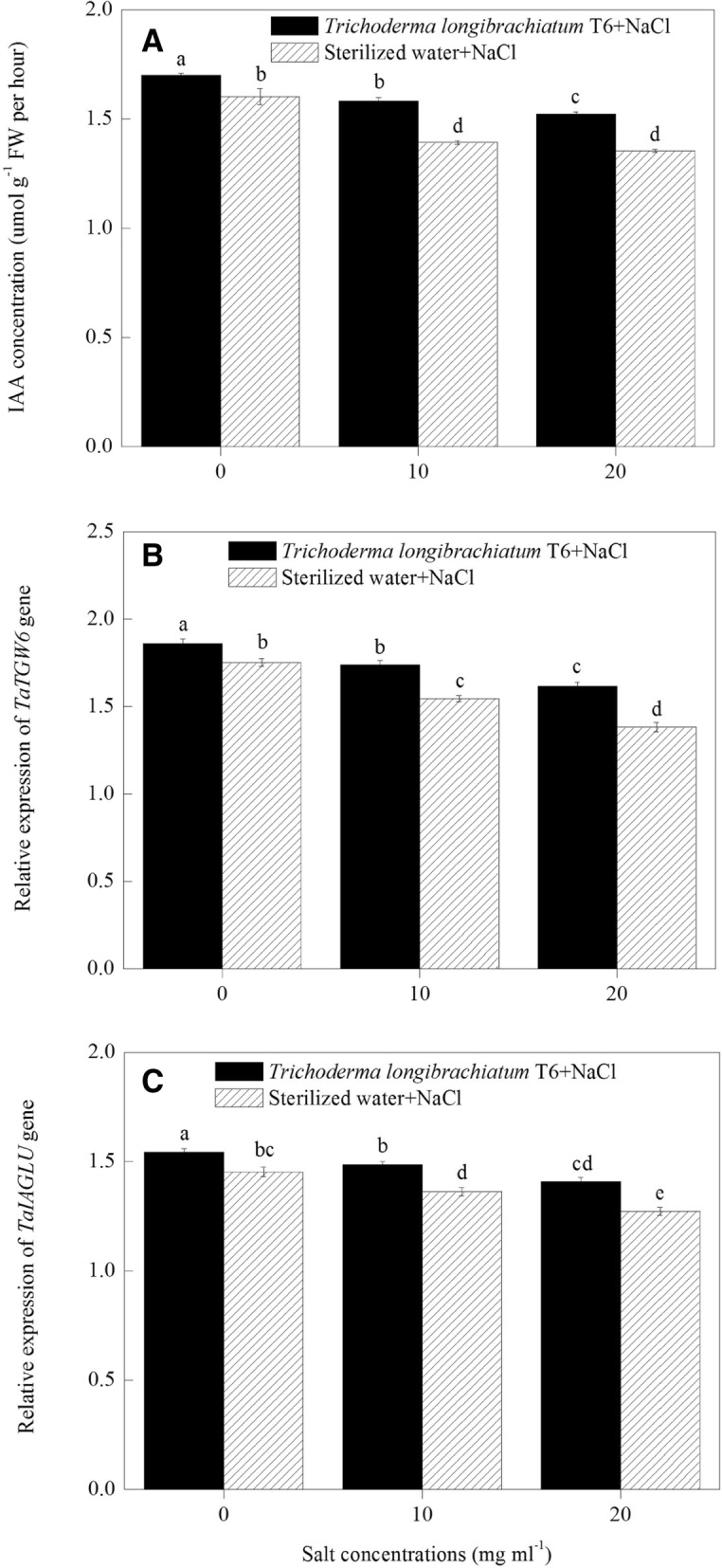
Effect of the strain of *Trichoderma longibrachiatum* T6 on (**a**) IAA production, and (**b**) the expression of *TaTGW6* and (**c**) *TaIAGLU* genes in wheat seedling roots under NaCl stress. The line bars represent the standard errors of the means. Different letters denote significant difference at the *P* < 0.05 level by Duncan’s new multiple range test (*n* = 12). In the three TL-6 treatments, wheat seeds were presoaked with the suspension of TL-6 spores for 12 h, whereas in the three sterile water treatments, wheat seeds were presoaked with sterile water only

Wheat seedlings with the IAA and ACC-deaminase producing strain of TL-6 treatment under NaCl stress increased the IAA concentration significantly in wheat seedlings roots (*P* < 0.05). At each of the three NaCl levels, the wheat seedlings treated with the IAA and ACC-deaminase producing strain of TL-6 significantly increased the level of IAA concentration compared with sterile water treatment; the IAA concentration was increased by 6% (0 mg ml^− 1^), 14% (10 mg ml^− 1^) and 13% (20 mg ml^− 1^) in wheat seedlings roots, respectively (*P* < 0.05). However, in the sterile water treatment, the IAA concentration from wheat seedlings roots was decreased by 13% at the 10 mg ml^− 1^ of NaCl concentration and by 16% at the 20 mg ml^− 1^ of NaCl stress, compared with 0 mg ml^− 1^ of NaCl concentration (*P* < 0.05) (Fig. 3a).

### Effect of TL-6 on the relative transcript level of IAA synthesis gene expression in wheat seedling

The IAA and ACC-deaminase producing strain of TL-6 treatment increased the IAA production genes expression significantly in wheat seedlings roots under NaCl stress (*P* < 0.05). Compared to 0 mg ml^− 1^ NaCl stressed plants in sterile water treatment, NaCl stress (10 and 20 mg ml^− 1^) decreased the transcript levels of the *TaTGW6* (Fig. 3b) and *TaIAGLU* (Fig. 3c) genes expression in sterile water treatment, but the transcript levels of the *TaTGW6* (Fig. 3b) and *TaIAGLU* (Fig. 3c) genes expression were up-regulated significantly in wheat seedlings roots treated with the IAA and ACC-deaminase producing strain of TL-6 under each of the three NaCl levels (*P* < 0.05). *TaTGW6* gene expression in wheat seedling roots was up-regulated under salt stress (0, 10 and 20 mg ml^− 1^) by 6, 13, and 17% (Fig. 3b), and *TaIAGLU* gene by 6, 9, and 11% (Fig. 3c), respectively, after treated with the IAA and ACC-deaminase producing strain of TL-6, compared to the sterile water treatment (*P* < 0.05).

### ACO and ACS activity in wheat seedling

**Fig. 4:**
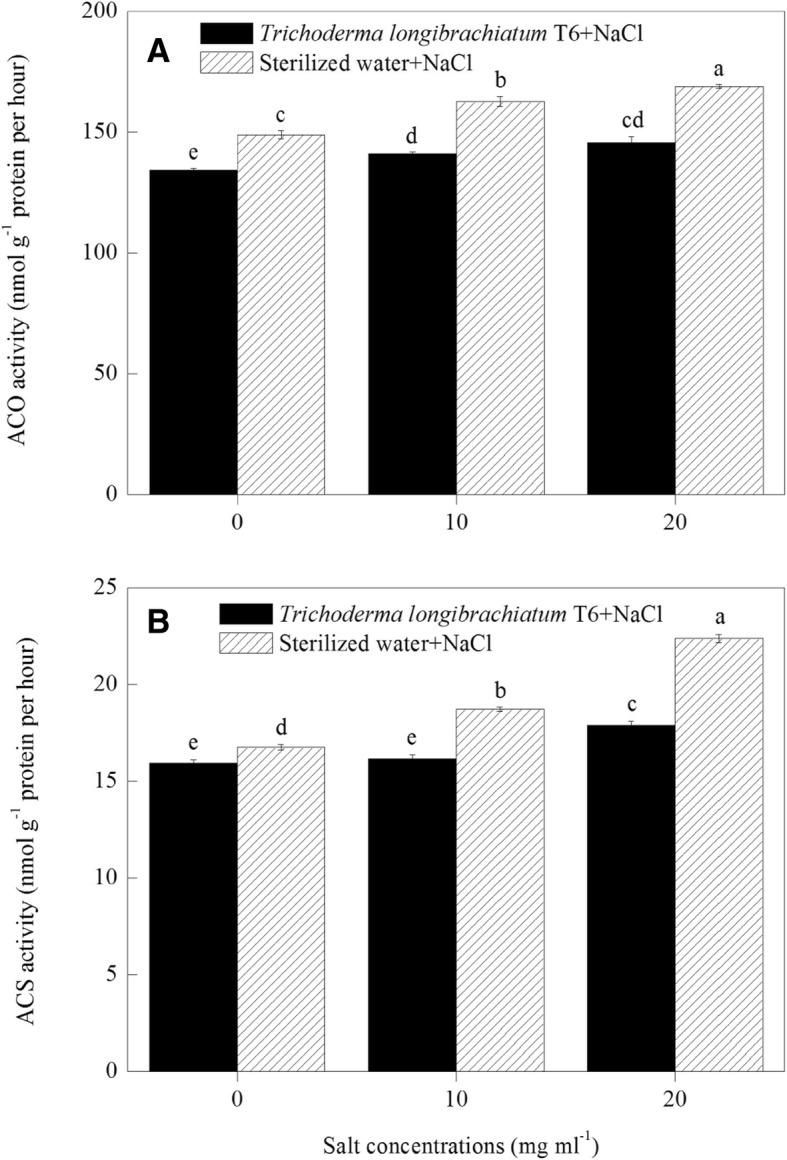
Effect of the strain of *Trichoderma longibrachiatum* T6 on (**a**) ACO activity and (**b**) the activity of ACS in wheat seedling roots under NaCl stress. The line bars represent the standard errors of the means. Different letters denote significant difference at the *P* < 0.05 level by Duncan’s new multiple range test (*n* = 12). The treatments are detailed in the footnote of Fig. 3

Wheat seedlings with the IAA and ACC-deaminase producing strain of TL-6 treatment under NaCl stress decreased the activity of ACO and ACS significantly in wheat seedlings roots (*P* < 0.05). Measured at Day 35, the seedlings treated with the IAA and ACC-deaminase producing strain of TL-6 decreased the activity of ACO by 10% at the NaCl concentration of 0 mg ml^− 1^, furthered to 13% at 10 mg ml^− 1^ and 14% at 20 mg ml^− 1^ (Fig. 4a), whereas the TL-6 treatment decreased the activity of ACS by 5, 14 and 20% (Fig. 4b), respectively, compared with sterile water treatment (*P* < 0.05). However, the activity of ACO and ACS in wheat seedlings roots treated with sterile water was increased significantly with the increase of NaCl concentrations from 0 to 20 mg ml^− 1^ (*P* < 0.05). The activity of ACO was increased by 9 to 13% (Fig. 4a) and the activity of ACS was increased by 12 to 34% (Fig. 4b) with the NaCl solution increasing from 10 to 20 mg ml^− 1^, compared with 0 mg ml^− 1^ of NaCl concentration under sterile water treatment (*P* < 0.05).

### ACC content and ethylene synthesis in wheat seedling

**Fig. 5:**
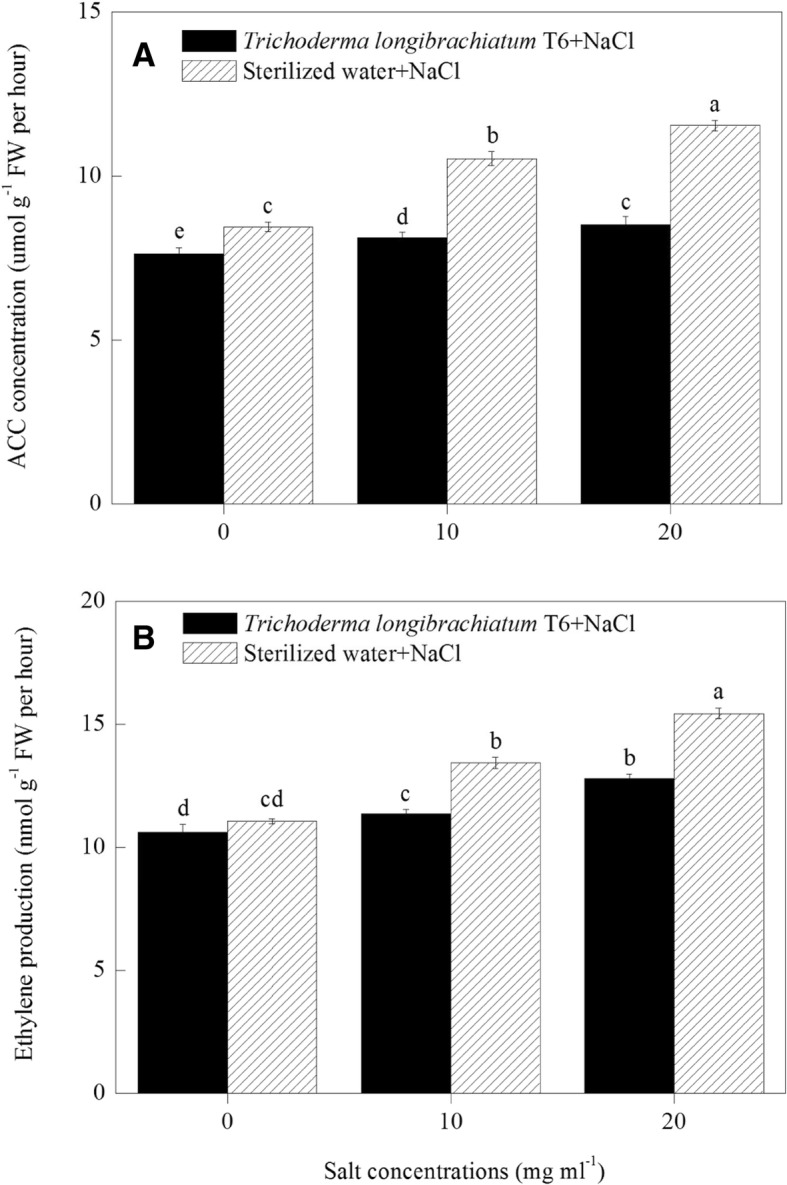
Effect of the strain of *Trichoderma longibrachiatum* T6 on (**a**) ACC content and (**b**) ethylene production in wheat seedling roots under NaCl stress. The line bars represent the standard errors of the means. Different letters denote significant difference at the *P* < 0.05 level by Duncan’s new multiple range test (*n* = 12). The treatments are detailed in the footnote of Fig. 3

ACC content and ethylene synthesis in wheat seedlings roots were determined under different levels of NaCl stress after the application of the IAA and ACC-deaminase producing strain of TL-6 or sterile water. The content of ACC in wheat seedlings roots significantly increased after treated with the NaCl solution increasing from 10 to 20 mg ml^− 1^ under sterile water treatment. In the wheat roots, the content of ACC was increased by 25 to 37%, compared with 0 mg ml^− 1^ of NaCl concentration under sterile water treatment (*P* < 0.05) (Fig. 5a). However, application of IAA and ACC-deaminase producing strain of TL-6 significantly decreased the content of ACC in the wheat seedlings roots under salt stress, compared with sterile water treatment. The content of ACC was decreased by 10% at the NaCl concentration of 0 mg ml^− 1^, furthered to 29% at 10 mg ml^− 1^ and 26% at 20 mg ml^− 1^, compared with sterile water treatment (*P* < 0.05). These results showed that the application of the IAA and ACC-deaminase producing strain of TL-6 decreased the content of ACC in wheat seedlings roots.

In addition, in the sterile water treatment, the ethylene production in wheat seedlings was 22% greater at 10 mg ml^− 1^ of NaCl concentration and was 40% greater at 20 mg ml^− 1^, compared with 0 mg ml^− 1^ of NaCl concentration (*P* < 0.05) (Fig. 5b). Regardless of the salt level, the wheat seedlings treated with the IAA and ACC-deaminase producing strain of TL-6 decreased the ethylene production significantly compared with the sterile water treatment. Averaged across the three (0, 10, 20 mg ml^− 1^) NaCl levels, the wheat seedlings treated with TL-6 decreased the ethylene production by 12% compared with sterile water treatment (*P* < 0.05) (Fig. 5b).

### Effect of TL-6 on the relative transcript level of ethylene synthesis gene expression in wheat seedling

**Fig. 6:**
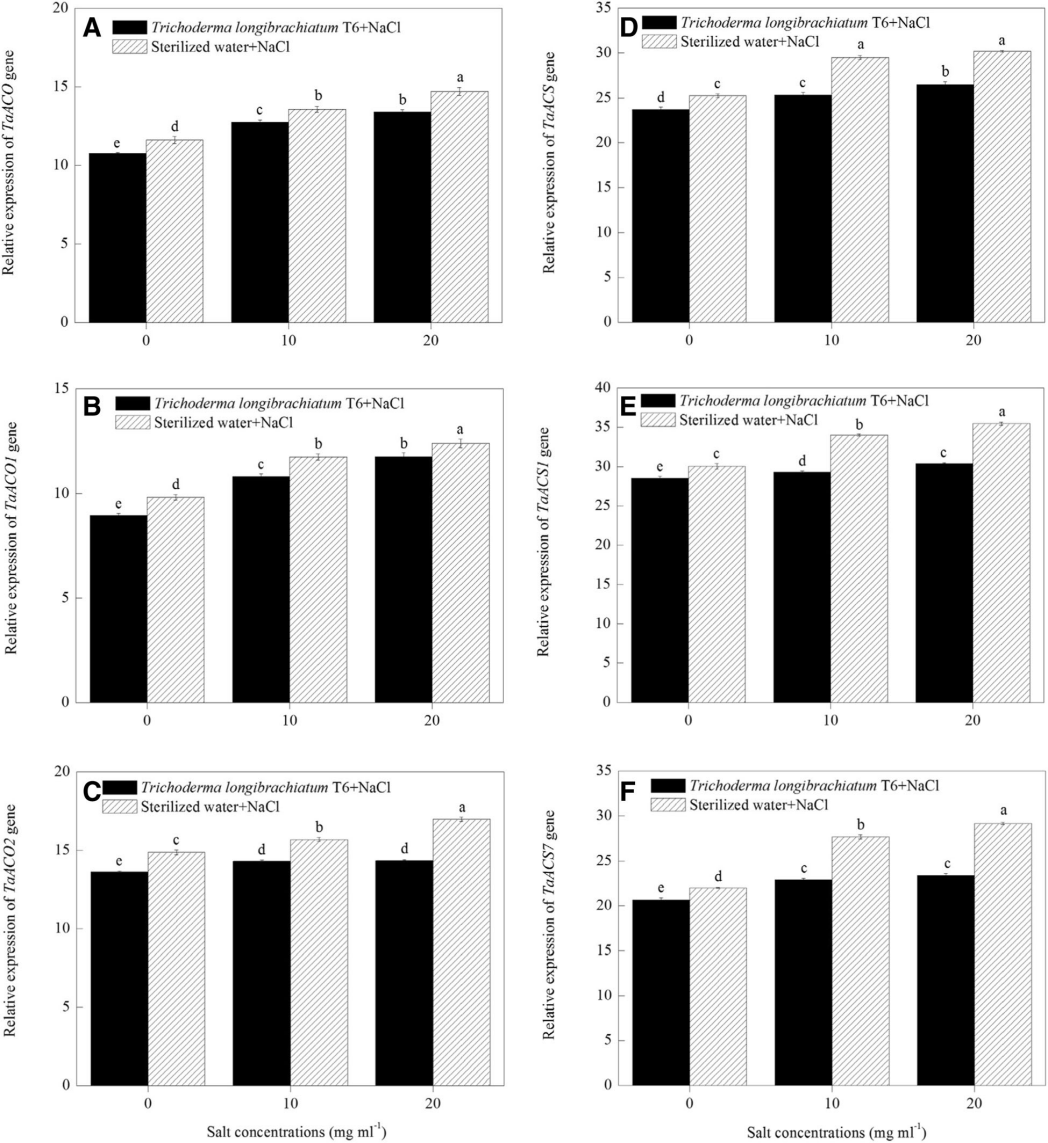
Effect of *Trichoderma longibrachiatum* T6 on the expression of (**a**) *TaACO*, (**b**) *TaACO1*, (**c**) *TaACO2*, (**d**) *TaACS*, (**e**) *TaACS1* and (**f**) *TaACS7* genes in wheat seedling roots under salt stress. The line bars represent the standard errors of the means. Different letters denote significant difference at the *P* < 0.05 level by Duncan’s new multiple range test (*n* = 12). The treatments are detailed in the footnote of Fig. 3

The TL-6 treatment decreased the transcriptional level of ethylene synthesis genes expression significantly in wheat seedlings roots under NaCl stress (*P* < 0.05). Compared to the control plants, there were higher levels of *TaACO* (Fig. 6a), *TaACO1* (Fig. 6b), *TaACO2* (Fig. 6c), *TaACS* (Fig. 6d), *TaACS1* (Fig. 6e) and *TaACS7* (Fig. 6f) genes expression in wheat seedlings roots after being induced by NaCl stress in the sterile water treatment. In contrast, at each of the three NaCl levels (0, 10, 20 mg ml^− 1^), the application of IAA and ACC-deaminase producing strain of TL-6 led to the transcript levels of *TaACO* (Fig. 6a), *TaACO1* (Fig. 6b), *TaACO2* (Fig. 6c), *TaACS* (Fig. 6d), *TaACS1* (Fig. 6e) and *TaACS7* (Fig. 6f) genes were down-regulated expression compared with sterile water treatment.

### Effect of TL-6 on Na^+^ and K^+^ concentration in wheat seedling under NaCl stress

**Fig. 7:**
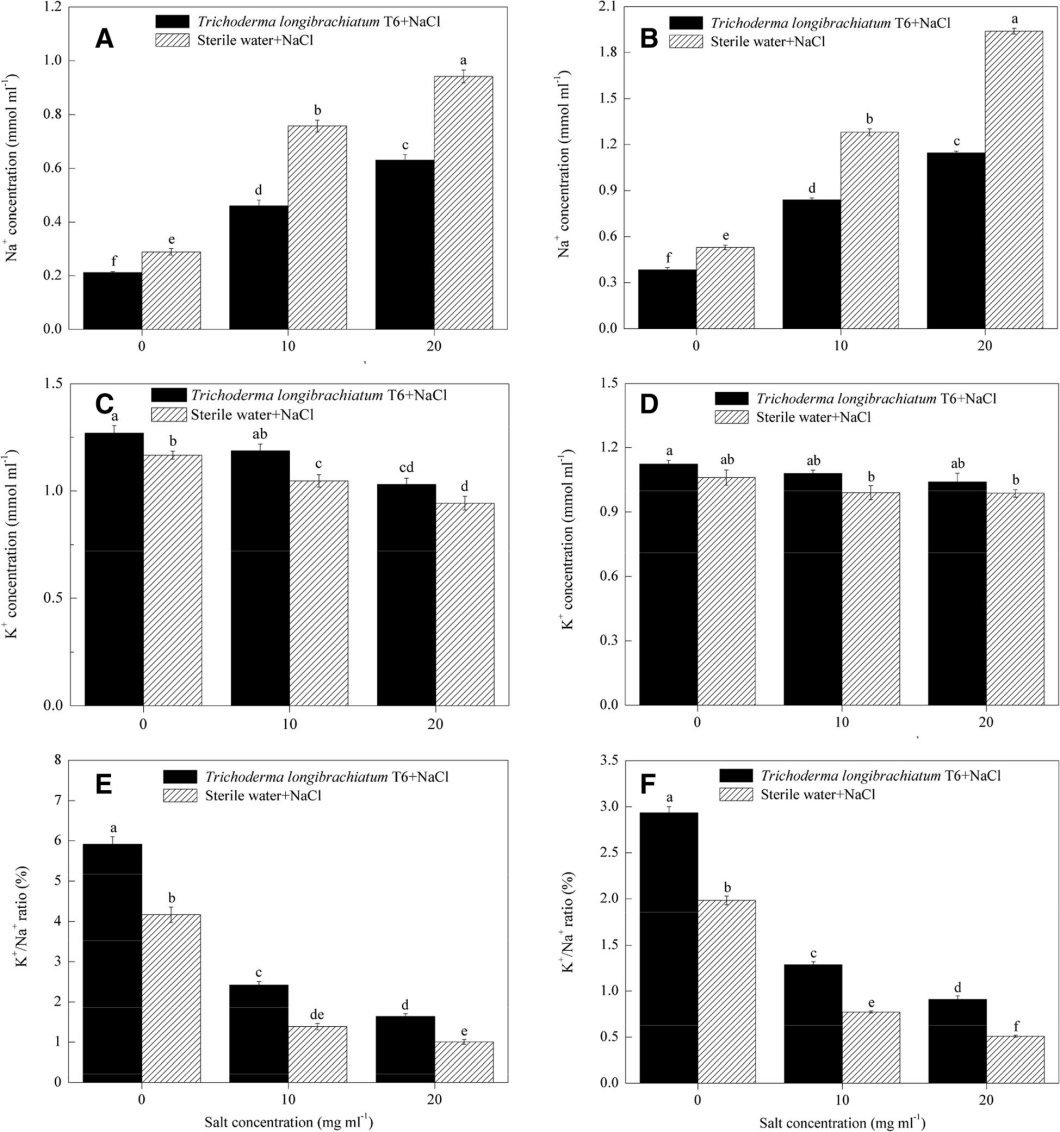
Effect of the strain of *Trichoderma longibrachiatum* T6 on Na^+^ (**a** and **b**) and K^+^ (**c** and **d**) concentration, and K^+^/Na^+^ ratio (**e** and **f**) in wheat seedling under NaCl stress. Where **a**, **c** and **e** represent Na^+^ and K^+^ concentration, and K^+^/Na^+^ ratio in the shoot of wheat seedling; **b**, **d** and **f** represent Na^+^ and K^+^ concentration, and K^+^/Na^+^ ratio in the root of wheat seedling. The line bars represent the standard errors of the means. Different letters denote significant difference at the *P* < 0.05 level by Duncan’s new multiple range test (*n* = 12). The treatments are detailed in the footnote of Fig. 3

The Na^+^ and K^+^ concentration in wheat seedling were measured at Day 35 under NaCl stress after the application of the IAA and ACC-deaminase producing strain of TL-6 or sterile water. Compared to the 0 mg ml^− 1^ NaCl stressed plants, the concentration of Na^+^ was significantly increased in wheat seedling shoots and roots under 10 and 20 mg ml^− 1^ NaCl stress, whereas the concentration of K^+^ and the ratio of K^+^/Na^+^ were decreased with increasing of salt concentrations in the sterile water treatment (*P* < 0.05) (Fig. 7). In contrast, significant differences were observed and detected between the sterile water and TL-6 treatments with respect to Na^+^ and K^+^/Na^+^ ratio in the shoots and roots of wheat seedling under 0, 10 and 20 mg ml^− 1^ NaCl stress. A significant decrease in Na^+^ concentration and increase in K^+^/Na^+^ ratio, and also slight increase in K^+^ absorption were observed in the shoots and roots after the application of the IAA and ACC-deaminase producing strain of TL-6 under 0, 10 and 20 mg ml^− 1^ NaCl stress in comparison to the sterile water treatment (*P* < 0.05). Pretreatment with the IAA and ACC-deaminase producing strain of TL-6 significantly decreased the Na^+^ concentration in shoots by 27% at the 0 mg ml^− 1^ of NaCl treatment, 39% at 10 mg ml^− 1^and 33% at 20 mg ml^− 1^ (Fig. 7a), and roots by 28, 34, and 41% (Fig. 7b), respectively; as well as the K^+^ concentration in roots was increased by 4, 6, and 8% (Fig. 7c) with 0, 10 and 20 mg ml^− 1^ NaCl stress, respectively, and also roots by 6, 8, and 5% (Fig. 7d), respectively (*P* < 0.05). Similarly, the ratio of K^+^/Na^+^ in the shoots of wheat seedling was increased by 43, 75, and 63% (Fig. 7e) with 0, 10 and 20 mg ml^− 1^ NaCl stress and also in roots were increased by 47, 66, and 79%, respectively (*P* < 0.05) (Fig. 7f).

### Effect of TL-6 on *SOS1* relative transcript level in wheat seedling

**Fig. 8:**
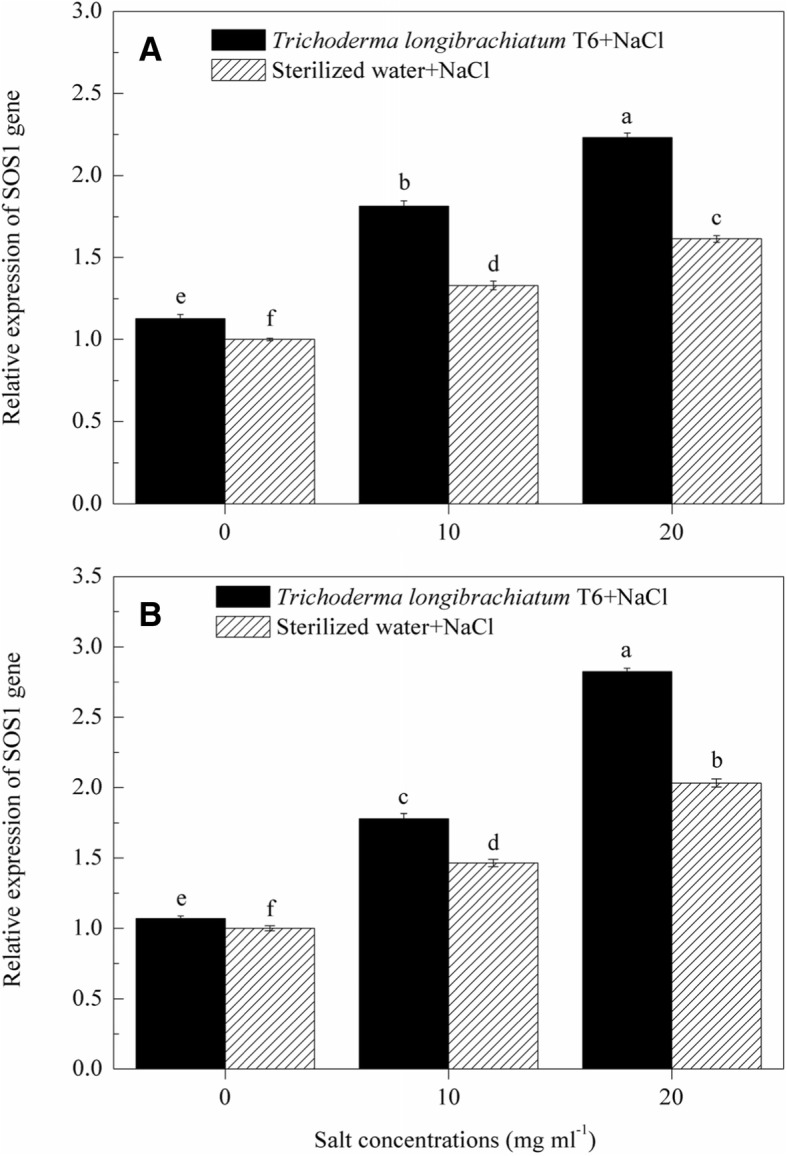
Effect of *Trichoderma longibrachiatum* T6 on the expression of *SOS1* gene in wheat seedlings shoot (**a**) and root (**b**) under salt stress. The line bars represent the standard errors of the means. Different letters denote significant difference at the *P* < 0.05 level by Duncan’s new multiple range test (*n* = 12). The treatments are detailed in the footnote of Fig. 3

Our results indicate that *SOS1* gene plays an important role in regulating the Na^+^ transportation under salt stress, and alleviating the Na^+^ damage effects in wheat seedling shoots and roots (Fig. 8). Compared to the 0 mg ml^− 1^ NaCl stressed plants, *SOS1* gene expression was up-regulated in wheat seedling shoots and roots under salt stress (10 and 20 mg ml^− 1^) in the sterile water treatment. At each of the three NaCl levels (0, 10, 20 mg ml^− 1^), the transcript level of *SOS1* gene in wheat seedling shoots and roots treated with the IAA and ACC-deaminase producing strain of TL-6 was significantly higher than those of sterile water treatment. *SOS1* gene expression in wheat seedling shoots was up-regulated under salt stress (0, 10 and 20 mg ml^− 1^) by 13, 36, and 38% (Fig. 8a), and roots by 7, 22, and 39% (Fig. 8b), respectively, after treated with the beneficial strain of TL-6, compared to the sterile water treatment (*P* < 0.05).

## Discussion

*Trichoderma* strains are free-living fungi in soil and can colonize plant roots and promote plant growth [4, 25]. A number of mechanisms for *Trichoderma* spp. promoting plant growth have been proposed [25], but there is little information available in regard to the mechanisms of *Trichoderma longibrachiatum* T6 (TL-6) promotes wheat growth and enhances plant tolerance to different levels of NaCl stress. The present study, through a series of in vitro and greenhouse experiments, determined the potential of TL-6 in tolerance to salt stress and the mechanisms of TL-6 promoting wheat seedling growth under various levels of salt stress. Our results showed that TL-6 promoted plant growth under saline condition largely through the increase of the activity of ACC-deaminase and the level of IAA production in TL-6 strain that induce the expression of genes encoding IAA as well as the level of IAA production, decrease the expression of genes encoding ethylene synthesis as well as the activity of ACO and ACS, and the content of ACC and the level of ethylene synthesis in wheat seedlings; alleviate the Na^+^ damage effects and enhance the transcriptional level of Na^+^/H^+^ antiporter gene expression in wheat plants. These improvements serve as the main mechanisms responsible for the IAA and ACC-deaminase producing strain of TL-6 promoting plant growth and enhancing salt tolerance in wheat.

High salinity decreases the growth of plants and the magnitude of this effect may be related to the interaction among the host, microbe, and the level of salt stress [22, 25]. Thus, it is of importance to determine whether different concentrations of NaCl solutions present a negative effect on the growth of TL-6. Our study showed that the low concentrations of NaCl had no negative effect on the growth of TL-6, and in fact a 10 mg ml^− 1^ of NaCl solution enhanced the TL-6 strain growth (both the diameter and the number of TL-6 spores) in comparison to those under non-saline condition. A NaCl concentrations greater than 20 mg ml^− 1^ significantly decreased the TL-6 strain growth, spores production and mycelia dry weight. This indicates that the effect of NaCl on the growth of TL-6 is in a dose-dependent manner, with high salinity inhibiting the growth, and low salinity promoting its growth [26]. In a study, Contreras-Cornejo et al. [27] found that salt stress decreased the growth of *Trichoderma* spp. in a dose-dependent manner, where the strains tolerated 8.8 mg ml^− 1^ of NaCl stress, but the growth of strains was decreased significantly with the salt concentration increased to 17.6 mg ml^− 1^. High salt concentrations may enhance the water potential of the substrate that reduces the growth of fungal colonies [28]. Also, high salt may affect cytoplasmic metabolic activity, such as intracellular proteins which may provide the extra osmotic potential to prevent plasmolysis [29]. Our results indicate that a dose of salt lower than 20 mg ml^− 1^ is adequate to determine the response of wheat plants to salt stress at the presence of *Trichoderma* spp.

In cucumber (*Cucumis sativus* L.), the use of *T. asperellum* Q1 strain promoted the plant growth due to the increased production of siderophore and auxin, and the enhanced activity of ACC-deaminase and phosphate solubilization [13]. However, little information is available regarding to the production of auxin and the activity of ACC-deaminase in TL-6 under salt stress. An unknown question was whether or not IAA and ACC-deaminase derived from the TL-6 strain play a role in alleviating salt stress in wheat. In the present study, we found that TL-6 did produce IAA and the quantity of IAA production was increased with the salt stress increased from 0 to 20 mg ml^− 1^ of salt concentration. Many other studies have also demonstrated that rhizosphere microorganisms can produce auxin alike signaling that promotes plant root branching and improves plant biomass production [4, 30–32]. Some of the rhizosphere microorganisms can help improve the fitness of plant-microbe interactions by producing IAA [33]. An added value from the present study is that the IAA production in the TL-6 under salt stress depends on the concentration of NaCl solution; a low concentration of 10 mg ml^− 1^ of NaCl stress increased IAA production significantly, and an increase of concentration to 20 mg ml^− 1^ had little additional effect on IAA production. In ours and other studies, the increased level of IAA production in the beneficial microorganisms may serve as an important signaling for plants to tolerate salt stress. The mechanisms of *T. asperellum* Q1 in alleviating the suppression effect of salt stress on cucumber growth involving in the ability to produce IAA, gibberellin (GA) and abscisic acid (ABA) both in the presence and absence of NaCl, and also the levels of endogenous IAA, GA and ABA in cucumber leaves were also changed correspondingly in pot experiments [34]. Interestingly, we also found that wheat seedlings treated with the IAA producing strain of TL-6 increased the level of IAA production significantly, as well as the expression level of two genes encoding IAA production markedly up-regulated in wheat seedling roots under salt stress condition. These findings indicate that the application of TL-6 strain significantly activated the IAA regulated genes expression that encoding IAA production significantly increased in wheat seedling roots to modulate plant growth under salt stress. Contreras-Cornejo et al. (2009), who demonstrated that the strain of *T. virens* Gv. 29–8 promotes *Arabidopsis* growth through auxin response pathway to modulate plant growth and activate auxin regulated gene expression [4]. However, to the best of our knowledge, this is the first report of TL-6 modulates wheat plant growth through the increased level of IAA production in TL-6 strain that induces the expression of genes encoding IAA synthesis as well as the level of IAA production in wheat seedlings roots under different levels of NaCl stress.

Some previous studies have also demonstrated that various biotic and abiotic stresses can cause an imbalance in ethylene biosynthesis [35–37]. The mechanisms for ethylene biosynthesis also have been reported that mainly including two main successive enzymatic reactions, (i) conversion of S-adenosylmethionine to 1-aminocyclopropane-1-carboxylic acid by ACS, which is generally considered as the rate-limiting step in ethylene biosynthesis, and (ii) conversion of ACC to ethylene by ACO to produce ethylene in various plant organs [38, 39]. In addition, it is common that ethylene is overproduced in plants under high salinity, and the presence of ACC-deaminase can reduce the negative consequence of ethylene on plant growth [40]. Similarly, heterologous expression of ACC-deaminase from *T. asperellum* can improve the growth performance of *Arabidopsis thaliana* under normal and salt stress conditions [41]. However, little is known about the mechanisms for the ACC-deaminase producing strain of TL-6 that promotes wheat seedlings growth and enhances plant tolerance to salt stress. In the present study, our results found that the increased activity of ACC-deaminase in TL-6 was observed under the concentrations of 10 and 20 mg ml^− 1^ of NaCl solutions. Wheat seedlings treated with the ACC-deaminase producing strain of TL-6 decreased the expression level of genes encoding ACS and ACO as well as the activity of ACO and ACS, and the content of ACC and the level of ethylene production significantly in wheat seedlings roots under salt stress condition. Similar report has been found that the strain *T. asperellum* T203 can produce ACC-deaminase that regulates the endogenous ACC level to reduce adverse effects of ethylene on canola (*Brassica napus* L.) growth [5, 42]; the strain *H. seropedicae* SmR1 unlike *A. brasilense* AbV5, presents a gene encoding the ACC-deaminase, which breaks ACC, the ethylene precursor in alpha-keto-butyric acid (AKB) and ammonium ion [43]; application of exogenous spermidine can reverse salinity-induced ethylene production by inhibiting the transcription and activity of ACS under salt stress [44]. Our results indicate that the promoted ACC-deaminase activity in TL-6 by decreasing the ethylene synthesis in wheat seedlings, which served as an important signal in promoting wheat seedling growth and enhancing plant tolerance to salt stress.

In addition, previous report showed that both IAA and ACC-deaminase can stimulate plant root elongation [45]. Similarly, Gao et al. (2018) reported that the species of *Pseudomonas putida* and *T. atroviride* can modulate the regulation of IAA and ethylene in the rhizosphere and within the roots to promote the development of the root system and of the tomato (*Solanum lycopersicum*) plant by their ability to produce and degrade IAA, and ACC-deaminase activity in general [46]. Grave et al. (2007) found that the phytohormone of IAA produced by the microbes that can modulate the synthesis of plant ethylene, such as inhibits the transformation of ACC into ethylene by decreasing the activity of ACO [47]. Although the regulation of IAA and ethylene in the rhizosphere or within the plant roots by the microbes have been previously reported, there is little information concerning the use of TL-6 enhanced the tolerance of wheat seedlings to salt stress at biochemical, physiological and molecular levels. Our findings suggest that the IAA and ACC-deaminase producing strain of TL-6 protects wheat plants from salt stress through the decrease of the expression level of genes encoding ACS and ACO as well as the activity of ACO and ACS, and further decreases the ACC and ethylene biosynthesis, as well as the increase of the expression level of genes encoding IAA as well as the concentration of IAA in wheat seedling roots to enhance wheat seedlings in response to salt stress.

Furthermore, a number of studies have been reported that plant cells under salt stress showed increased toxic level of cellular Na^+^ and restricted absorption of macroelement K^+^, which causes a rapid reduction of K^+^/Na^+^ ratio in cytoplasm [48] and disturbs the intracellular ionic homeostasis in plant cells [49]. Therefore, the decreasing of Na^+^ accumulation and maintaining a high K^+^/Na^+^ ratio in pant tissues are considered as important mechanisms which response for plant growth and tolerance to salt stress [49, 50]. In the present study, our results revealed that the Na^+^ concentration in wheat seedlings significantly increased and that the ratio of K^+^/Na^+^ and K^+^ concentration were decreased under salt stress. Interestingly, application of the IAA and ACC-deaminase producing strain of TL-6 alleviates the ion-specific toxicity significantly by decreasing cellular accumulation of Na^+^ and increasing the ratio of K^+^/Na^+^ in both shoots and roots of wheat seedlings under NaCl stress. Several reports have demonstrated that the role of beneficial soil bacteria in improving plant tolerance to drought and salinity stress [51–53]. Zhang et al. (2014) found that the beneficial rhizobacterium *B. subtilis* (GB03) improved salt tolerance of wheat by decreasing Na^+^ accumulation and increasing K^+^/Na^+^ ratio [54]. Singh and Jha (2016) reported that application of an ACC-deaminase-producing halophilic bacterium *Serratia* sp. SL-12 decreased the levels of Na^+^ by 65% and increased the K^+^ absorbtion by 39% under salt stress [55]. Additionally, Contreras-Cornejo et al. (2014) demonstrated that *Trichoderma* spp. improve growth of *Arabidopsis* seedlings under salt stress through enhanced root development, osmolite production, and Na^+^ elimination [27]. However, our present study showed that the IAA and ACC-deaminase producing strain of TL-6 significantly decreased Na^+^ accumulation and increased K^+^/Na^+^ ratio, and slightly increased K^+^ absorption in wheat, which in line with the results from Niu et al. (2016), who reported that the strain of GB03 significantly decreased whole plant Na^+^ content, restricted K^+^ absorption, and therefore, increased K^+^/Na^+^ in both shoots and roots [56]. Thus, our results indicate that the strain of TL-6 enhanced salt tolerance in wheat seedlings through a reduction of Na^+^ concentration and increasing of K^+^/Na^+^ ratio, which play significant role in maintaining ionic homeostasis and minimizing toxic ionic effects on wheat seedlings [55].

Additionally, the regulation of ions within the cell cytosol of plants through the plasma membrane and endomembrane transporters are considered as an indispensable component of plant growth and adaptation to salinity [57]. The extra Na^+^ ions in cytosol can be exported to extracellular through Na^+^/H^+^ exchangers localized in the plasma membrane and to vacuole under salt stress [58]. Several important plasma membrane exchangers, such as the salt overly sensitive (SOS) pathway is essential for salt stress tolerance and maintaining ion homeostasis in the cytoplasm [59]. Among the SOS proteins, SOS1 (a plasma membrane Na^+^/H^+^ antiporter) playing a key role in the extrusion of excess toxic Na^+^ from cells [60]. Similar study indicated that SOS1, a highly conserved protein in mediating Na^+^ transportation in *Arabidopsis* and *Puccinellia tenuiflora* have important functions in regulating the cytosolic Na^+^ efflux [61]. Our results indicated that Na^+^/H^+^ antiporter gene *SOS1* expression was up-regulated with increasing of salt stress. Additionally, the transcript levels of *SOS1* gene treated with TL-6 were significantly higher than those of the other groups, which is consistent with the improved salt tolerance and reduced Na^+^ accumulation in shoots and roots of wheat seedlings. Also, our results indicate that *SOS1* gene plays an important role in regulating the Na^+^ transportation under high salinity, alleviating the Na^+^ damage effects, which in line with the Na^+^/H^+^ exchangers in plants synergically function to cope with the extra cytosolic Na^+^ when plants are exposed to a high-salinity condition [58].

## Conclusions

Salt stress decreased the growth of wheat seedlings and the negative effect was alleviated significantly with the supplement of the beneficial microorganism *Trichoderma longibrachiatum* T6 (TL-6). The beneficial role of TL-6 was reflected by the increased ACC-deaminase activity and IAA production in TL-6 to modulate the synthesis of ethylene and IAA, Na^+^ and the ratio of K^+^/Na^+^ in wheat seedlings that promote plant growth and enhance plant tolerance to salt stress; these functions were in a salt concentration dose-dependent manner. Our results revealed two possible mechanisms: (i) the promoted ACC-deaminase activity and increased IAA production in TL-6 by increasing the IAA concentration and decreasing the ethylene synthesis in wheat seedlings, which served as an important signal in alleviating the negative effect of salt stress on wheat seedlings; and (ii) the promoted ACC-deaminase activity and increased IAA production in TL-6 by minimizing the ionic toxicity in wheat seedlings in response to salt stress.

## Materials and methods

Experiments were carried out at the Gansu Provincial Biocontrol Engineering Laboratory of Crop Diseases and Pests. All treatments in the experiments described below had six replicates and each experiment was repeated twice over time, unless otherwise indicated.

### Fungal material

*Trichoderma longibrachiatum* T6 (TL-6) was isolated from a rhizisphere saline-soil of a forest site nearby Tianshui, Gansu. The TL-6 strain was cultured on potato dextrose agar media for 5 to 6 days at 25 °C. The spore concentration in the suspension was prepared according to the procedure described previously by Zhang et al. (2014) [62]. The final spore concentration of TL-6 was adjusted to 1 × 10^8^ spores ml^− 1^.

### Seeds treatment

The wheat (*Triticum aestivum* L.) cultivar ‘Yongliang 4’ provided by Gansu Academy of Agricultural Sciences was used in all the experiments. No any permissions were necessary to collect the plant samples. Wheat seeds with a uniform size were surface-sterilized with 1% NaOCl for 5 min and then with 95% (*v*/v) ethanol for 5 additional minutes. After disinfection, all the seeds were rinsed with sterile water, and then were soaked in TL-6 spore suspension at the concentration of 1 × 10^8^ spores ml^− 1^ for 12 h. The control seeds were soaked in sterile water for 12 h.

### Effect of NaCl stress on colony diameter, spores production and mycelia weight of TL-6 strain

The different amounts of NaCl crystal (0, 0.2, 0.4, 0.6, 0.8 and 1.0 g) were added into each 20 ml of sterilized potato dextrose agar media at 50 °C, making six different concentrations of NaCl at 0, 10, 20, 30, 40 and 50 mg ml^− 1^, respectively. The solutions were placed on Petri dishes after 30 s of shaking. TL-6 mycelia discs (5 mm) of active culture were transferred to the centre of potato dextrose agar media plates with different concentrations of NaCl solutions, and were incubated at 25 °C with supplemental day/night lighting of 16/8 h. Potato dextrose agar media inoculated with TL-6 mycelia disc but not with NaCl solution were considered as the control (0 mg ml^− 1^). Two days after inoculation, the colony diameter was measured daily, and the number of spore production was determined at Day 7 of incubation.

Flask culture experiments were performed using 150 ml of flasks that each contained 60 ml of potato dextrose broth media and different amounts of NaCl crystal (0, 0.6, 1.2, 1.8, 2.4 and 3.0 g), and then inoculated with 1 ml of spore suspension of TL-6 (1 × 10^8^ spores ml^− 1^). The potato dextrose broth media inoculated with an equal amount of spore suspension of TL-6 (1 ml) but not with NaCl solution were considered as the control (0 mg ml^− 1^). The fermentation media were incubated at 25 °C for 5 days with shaking at 180 rpm min^− 1^. At Day 5, the fermentation was filtered using sterilized filer for three times, the mycelia were collected from the filter, oven dried at 80 °C for 30 min, and weighed for mycelia dry weight.

### IAA production in TL-6

For the determination of the production of IAA in TL-6, 1 ml of spore suspension of TL-6 (1 × 10^8^ spores ml^− 1^) was added to 100 ml of potato dextrose broth media supplemented with L-tryptophan at 100 mg l^− 1^ in the NaCl concentration of 0, 10 and 20 mg ml^− 1^. The fermentation broth was grown in shaker at 180 rpm min^− 1^ for 5 days at 28 °C, centrifuged at 10, 000 g for 20 min at 4 °C, and the culture was filtrated through a Whattman Paper No.3 filter and followed by filtration through 0.22 μm Millipore membranes. IAA concentration was determined according to the method of Salkowski reagent [63]. The concentration of IAA was determined by comparison with a standard curve prepared in an IAA standard curve.

### ACC-deaminase activity determination in TL-6

For the determination of the ACC-deaminase activity of TL-6 under salt stress, 1 ml of spore suspension of TL-6 (1 × 10^8^ spores ml^− 1^) was inoculated in 60 ml of synthetic medium [64] in the 0, 10 and 20 mg ml^− 1^ of NaCl solutions. The culture was grown at 28 °C with shaking at 180 rpm min^− 1^ for 5 days. At Day 5 of incubation, the mycelia were collected and suspended in 2.5 ml of Tris buffer (0.1 M, pH 8.5), and homogenized for 30 s. Afterwards, 25 μl of toluene was added to a 200 μl aliquot and vortexed vigorously for 30 s, and then 20 μl of 0.5 M solution of ACC was added in the mixtures (no ACC added in the control). After an incubation period at 30 °C for 15 min, 1 ml of 0.56 N HCl was added and the reaction mixtures were centrifuged at 10, 000 g for 10 min, and then 1 ml of the supernatant was mixed with 800 μl of 0.56 N HCl and 300 μl of 2, 4-dinitrophenylhydrazine. Thereafter, 2 ml of 2 N NaOH was added to the mixtures after an incubation period at 30 °C for 30 min. ACC-deaminase activity was evaluated quantitatively by measuring the amount of a-ketobutyrate produced by the deamination of ACC according to the method of Viterbo et al. [5], and was expressed as μmol a-ketobutyrate mg^− 1^ protein h^− 1^.

### Effect of IAA and ACC-deaminase producing strain of TL-6 on wheat seedling tolerance to NaCl stress in greenhouse

Wheat seeds (80 seeds) with a uniform size were planted in 10-cm diameter pots that contained 300 g of sterilized soil. A total of 50 seedlings per pot were kept through thinning at Day 12 after emergence. The experiments included two group treatments: (i) wheat seeds were soaked with the spore suspension of TL-6 and inoculated at 0, 10 and 20 mg ml^− 1^ of NaCl concentrations, and (ii) wheat seeds were soaked with sterile water and inoculated at 0, 10 and 20 mg ml^− 1^ of NaCl concentrations. Each of the NaCl-treated pots was irrigated with 25 ml of NaCl solution whereas the 0 mg ml^− 1^ of NaCl concentration treatment was irrigated with 25 ml of sterile water. Plants were grown in a greenhouse (25 °C) with supplemental day/night lighting of 16/8 h, and each pot was irrigated with 200 ml of sterile distilled water at regular intervals. The seedlings biochemical, physiological and molecular parameters were determined at Day 35.

### Extraction and determination of IAA production in wheat seedling under NaCl stress

For the determination of the concentration of IAA in wheat seedlings, roots samples (1 g) of 35-day-old wheat seedlings were frozen immediately and then homogenized with a mortar and pestle using 80% methanol. The pulverized mixture was stirred overnight at 4 °C, and the impurities were then removed by centrifuging at 10, 000 g for 20 min. The supernatant was filtrated and used to analyze the level of IAA production by high performance liquid chromatography [65].

### Assay of ACO and ACS activity in wheat seedling under NaCl stress

For the determination of the activity of ACO and ACS, wheat seedling roots samples of 1 g were frozen immediately and ground to a fine powder, and then added to 5.0 ml of an extraction buffer. Thereafter, the homogenate was centrifuged at 12, 000 g for 10 min and then the supernatant was used for ACO and ACS activity assay.

The activity of ACO was assayed according to the procedure described previously by Kato et al. (2000) with some modifications [66]. The purified supernatant was incubated in 2 ml reaction medium for 1 h at 30 °C, and then a sample of 2 ml gas was collected and used to determine the ethylene level on a gas chromatograph. The activity of ACO was determined as the amount of ethylene converted from ACC during the reaction period, and expressed as nanomoles ACC per gram protein per hour.

The activity of ACS was assayed according to the procedure described previously by Fan et al. (1998) with some modifications [67]. ACS activity was measured by incubating 2.0 ml of purified supernatant in a reaction mixture at 30 °C for 1 h. Thereafter, one milliliter of headspace gas sample was collected and injected into a gas chromatograph for ethylene assay. ACS activity was determined as the amount of ethylene converted from SAM during the reaction period, and expressed as nanomoles ACC per gram protein per hour.

### Determination of ACC content and ethylene synthesis in wheat seedling under NaCl stress

ACC extraction was extracted by a solid-phase extraction procedure according to the method described by Madhaiyan et al. (2007) [68]. Wheat seedling roots samples of 1 g were frozen in liquid nitrogen and ground to a fine powder, and then 1 ml of the gaseous portion was taken and assayed for ethylene synthesis by gas chromatography. ACC content was expressed as micromoles per gram fresh weight per hour [69].

Ethylene level was determined following the method of Yamauchi et al. (2014) with some modifications [70]. Roots samples of wheat seedlings (1 g) were placed in a container and ground to a fine powder with saturated sodium chloride solution. One milliliter of collected gas sample was used to measure the ethylene level by gas chromatography. Ethylene production was expressed as nanomoles per gram fresh weight per hour.

### Determination of Na^+^ and K^+^ concentration in wheat seedling under NaCl stress

For the determination of Na^+^ and K^+^ concentrations in plant tissues, wheat seedlings in each treatment were thoroughly washed three times with deionized water to remove surface salts, and then dried with absorbent paper. The shoots and roots were separated and oven dried at 65 °C for 2 days. The dried shoots and roots of 0.4 g were extracted with 20 ml of 100% HNO_3_ for 24 h, respectively, followed by incubation at 90 °C for 2 h. Thereafter, the digested samples and the solutions were filtered, and then the filtrates were diluted with sterile water to 10-fold. The concentrations of K^+^ and Na^+^ were determined by an atomic absorption spectrophotometry [52, 54].

### Total RNA extraction and first strand cDNA synthesis

Total RNA was extracted from the wheat seedlings of 35 days of old (200 mg sample) in all treatments including those treated with the IAA and ACC-deaminase producing strain of TL-6 or sterile water under different concentrations of NaCl solutions (0, 10 and 20 mg ml^− 1^). The extraction was conducted by following the manufacturer′s instruction of Tiangen RNA Simple Total RNA Kit (Tiangen Biotechnology, Beijing, China). The first-strand cDNA was synthesized according to the procedure described previously by Zhang et al. (2016) [24].

### Quantitative real-time PCR (qRT-PCR) analysis

Genes encoding ethylene and IAA synthesis, and Na^+^/H^+^ antiporter were identified in wheat seedlings after treated with the IAA and ACC-deaminase producing strain of TL-6 or sterile water under different concentrations of NaCl solutions. qRT-PCR was performed using a SYBR Premix Ex Taq kit (Takara Biotechnology, Dalian, China) following the manufacturer′s instruction. Specific primer for each gene (*TaTGW6*, *TaIAGLU*, *TaACO*, *TaACO1*, *TaACO2*, *TaACS*, *TaACS1*, *TaACS7*, *SOS1* and *Actin* genes) was designed according to the EST sequences of wheat in NCBI [46, 65, 71] using Primer Express 5.0 software that amplifies the target genes (Table 2). The *Actin* gene of wheat was used as an internal control. The relative expression of *TaTGW6*, *TaIAGLU*, *TaACO*, *TaACO1*, *TaACO2*, *TaACS*, *TaACS1*, *TaACS7* and *SOS1* genes was determined using the method of 2^-ΔΔCt^ [72]. All treatments had six replicates and was repeated twice, thus, the gene expression was the average value of twelve independent replicates.

**Table 2:** DNA sequences of qRT-PCR primers for the determination of the level of ethylene and IAA synthesis gene expression in wheat seedlings

Genes	Premiers sequence (5′-3′)
*TaTGW6*	F: CACCTCGTGGTCGCATCT
	R: ATCTGGGTAGCCCGGCAG
*TaIAGLU*	F: CGTGTTCGCGCTCAGCCAGT
	R: CGAGGGACGCGAAGCTGCCG
*TaACO*	F: CCTACCCGAGGTTCGTGTT
	R: CTCCTTGGCCTCGAACTTGT
*TaACO1*	F: TCCCAGGTTTGGAGTTTCTG
	R: ATAGATAGGCGGCTCCCATT
*TaACO2*	F: CCTACCCGAGGTTCGTGTT
	R: CTCCTTGGCCTCGAACTTGT
*TaACS*	F: GATCTCCATGGTCTGGTCGT
	R: CTCTTCTCGTGGATGGACCT
*TaACS1*	F: GAATTCGAT GGTGAGCCAAGT
	R: AGCGCGTGGGGGTTCTTCT
*TaACS7*	F: GAGGTGAAGCTCAACATCTCG
	R: TGTTCTTGCTGCGTTGACAT
*Actin*	F: AGCCATACTGTGCCAATC
	R: GCAGTGGTGGTGAAGGAGTAA

Note: *F* represents forward, *R* represents reverse

### Statistical analysis

A factorial design was applied to all the experiments in the study. The factors tested in each experiment included TL-6 or sterile water treatments at the different levels of salt stress. Each experiment had six replications and was repeated twice over time. The data were subject to ANOVA using SPSS Version 16.0 (SPSS Inc., Chicago, IL). Preliminary analysis showed that there were no significant interactions between the two runs and treatments, and thus the data from the two runs of experiments were pooled together in the ANOVA. The significance between the treatments was considered at the level of *P* < 0.05. Duncan’s new multiple range test values were computed using standard error and T-values of adjusted degrees of freedom.

## Abbreviations

ABA: abscisic acid
ACC: 1-aminocyclopropane-1-carboxylate
ACC-deaminase: 1-aminocyclopropane-1-carboxylate-deaminase
ACO: ACC oxidase
ACS: ACC synthase
FW: Fresh weight
GA: gibberellin
IAA: Indole acetic acid
SAM: S-adenosylmethionin
SOS: Salt overly sensitive
TL-6: *Trichoderma longibrachiatum* T6
